# Dopamine-Mediated Major Depressive Disorder in the Neural Circuit of Ventral Tegmental Area-Nucleus Accumbens-Medial Prefrontal Cortex: From Biological Evidence to Computational Models

**DOI:** 10.3389/fncel.2022.923039

**Published:** 2022-07-22

**Authors:** Yuanxi Li, Bing Zhang, Xiaochuan Pan, Yihong Wang, Xuying Xu, Rubin Wang, Zhiqiang Liu

**Affiliations:** ^1^Institute for Cognitive Neurodynamics, School of Mathematics, East China University of Science and Technology, Shanghai, China; ^2^Department of Neurology, David Geffen School of Medicine, University of California, Los Angeles, Los Angeles, CA, United States; ^3^Department of Anesthesiology, Shanghai First Maternity and Infant Hospital, Tongji University School of Medicine, Shanghai, China; ^4^Clinical and Translational Research Center, Shanghai First Maternity and Infant Hospital, Tongji University School of Medicine, Shanghai, China; ^5^Anesthesia and Brain Function Research Institute, Tongji University School of Medicine, Shanghai, China

**Keywords:** major depressive disorder, NAc-mPFC-VTA circuit, dopamine, Hodgkin-Huxley (HH) model, dynamical receptor binding model

## Abstract

Major depressive disorder (MDD) is a serious psychiatric disorder, with an increasing incidence in recent years. The abnormal dopaminergic pathways of the midbrain cortical and limbic system are the key pathological regions of MDD, particularly the ventral tegmental area- nucleus accumbens- medial prefrontal cortex (VTA-NAc-mPFC) neural circuit. MDD usually occurs with the dysfunction of dopaminergic neurons in VTA, which decreases the dopamine concentration and metabolic rate in NAc/mPFC brain regions. However, it has not been fully explained how abnormal dopamine concentration levels affect this neural circuit dynamically through the modulations of ion channels and synaptic activities. We used Hodgkin-Huxley and dynamical receptor binding model to establish this network, which can quantitatively explain neural activity patterns observed in MDD with different dopamine concentrations by changing the kinetics of some ion channels. The simulation replicated some important pathological patterns of MDD at the level of neurons and circuits with low dopamine concentration, such as the decreased action potential frequency in pyramidal neurons of mPFC with significantly reduced burst firing frequency. The calculation results also revealed that NaP and KS channels of mPFC pyramidal neurons played key roles in the functional regulation of this neural circuit. In addition, we analyzed the synaptic currents and local field potentials to explain the mechanism of MDD from the perspective of dysfunction of excitation-inhibition balance, especially the disinhibition effect in the network. The significance of this article is that we built the first computational model to illuminate the effect of dopamine concentrations for the NAc-mPFC-VTA circuit between MDD and normal groups, which can be used to quantitatively explain the results of existing physiological experiments, predict the results for unperformed experiments and screen possible drug targets.

## Introduction

Major depressive disorder (MDD) is a serious psychiatric disorder, with an increasing incidence in recent years (Liu and Thompson, 2017). At this stage, scientists in different fields have studied the causes of MDD from environmental, genetic, psychological, and social factors, but the mechanisms are still not clear (Kato and Serretti, 2010). From the perspective of neuroscience, the attack of MDD is usually accompanied by abnormal brain regions or neural circuits’ dysfunctions. Particularly, the experimental results reveal that the limbic system and the dopaminergic pathways play a very important role in the pathogenesis of MDD *via* modulating emotion and reward (Nestler and Carlezon, 2006; Russo and Nestler, 2013; Belujon and Grace, 2017). In those regions, the dopaminergic pathway consisting of the midbrain ventral tegmental area (VTA), nucleus accumbens (NAc), and medial prefrontal cortex (mPFC) are responsible for regulating the release of the neurotransmitter dopamine (DA), which can excite the brain to generate positive emotions and make a decision (Opmeer et al., 2010). A low level of dopamine can cause glumness and emotional indifference, and it is the important pathogenesis of MDD, as well as Alzheimer’s disease (Wolfe et al., 1990). Thus, the study of abnormal VTA-NAc-mPFC neural circuits has become a hot topic for exploring the causes of MDD (Russo and Nestler, 2013).

The anatomical evidence shows that in the VTA-NAc-mPFC pathway, the VTA, consisting mainly of dopaminergic neurons responsible for the synthesis and release of dopamine, is projected to other brain regions (including the mPFC and NAc) *via* synaptic connections (Beier et al., 2015). The mPFC consists mainly of excitatory pyramidal neurons and inhibitory interneurons (including parvalbumin/PV, somatostatin/Som, calbindin/CB, and other subtypes of interneurons) (Kelsom and Lu, 2013). The pyramidal neurons release glutamate, which causes excitatory postsynaptic potentials (EPSPs) to occur in the VTA, mPFC, and NAc regions and excite their membrane potentials. The interneurons mainly release GABA to inhibit membrane potentials of pyramidal neurons *via* inhibitory postsynaptic potentials (IPSPs), thus maintaining the stability of the neural network (Russo and Nestler, 2013). The NAc consists mainly of dopamine D1-/D2-type GABAergic medium spiny neurons (MSNs) and interneurons (Chen et al., 2019; Bjerke et al., 2021), which receive excitatory glutamate from PFC pyramidal neurons and release GABA to inhibit VTA dopaminergic neurons. That is the most important source of GABA received by VTA dopaminergic neurons. NAc interneurons have a similar role to that of mPFC interneurons and are responsible for regulating the stability of the membrane potentials in NAc (Russo and Nestler, 2013; Francis et al., 2015; Francis and Lobo, 2017; McCullough et al., 2021).

It has been reported that MDD rats showed lower levels of dopamine concentrations and metabolic rates in both the NAc and mPFC regions compared with normal rats (Tanda et al., 1994; De La Garza and Mahoney, 2004). This can affect the membrane potential. Some studies showed that the firing frequency of pyramidal neurons in mPFC was significantly reduced in MDD mice, along with a decrease in burst firing frequency (Onn et al., 2006). But the firing rates of NAc D2-MSNs in MDD mice did not change significantly whether it had quinpirole administration or not (a D2-like receptor agonist) in each group (Cepeda et al., 2008; Liu et al., 2014). The abnormal dopamine concentration can also lead to abnormal changes in the kinetic properties of some ion channels. For example, it was found that in PFC pyramidal neurons, dopamine can bind to D1 receptors and then activate an intracellular calcium pathway to reduce the excitatory of a slowly inactivating potassium current channel (KS) (Yang and Seamans, 1996; Yang et al., 1996). Meanwhile, regarding the D2-type MSNs of NAc, dopamine can bind to D2 receptors and not only excite the slow A-type sodium channel (KAs) directly, but also modulate the calcium channel to indirectly increase the excitability of the inwardly rectifying sodium channel (KIR) (Perez et al., 2006; Cepeda et al., 2008; Lindroos et al., 2018). Furthermore, although there was some evidence that dopamine can directly modulate synaptic receptors, such as AMPAR, NMDAR, and GABA_*a*_R, to change the membrane potentials, it was not sure whether this was a direct factor (Gao and Goldman-Rakic, 2003; Onn et al., 2006), as excitation-inhibition balance might be an equal role.

However, few studies worked on the mechanism to explain how dopamine concentration dynamically regulates this neural network by affecting ion channels and synaptic activities, since it was too difficult to perform multi-channel measurements simultaneously for dopamine concentration, membrane potentials, ion channels, and synaptic activities in the experiment. Therefore, we considered building a biological neural network model to explain these neuronal and network activities.

Although there were some computational models for MDD, almost all of them focused on electroencephalogram (EEG) signals (Čukić et al., 2020; Saeedi et al., 2021) and functional connectomes of functional MRI (fMRI) (Yan et al., 2019; Wang et al., 2020; Macpherson et al., 2021; Tokuda et al., 2021) but not on the cellular level. Therefore, we cannot use these models to explain the cellular mechanisms for MDD.

We have studied the abnormal firing patterns with ion channels in the individual D2-MSN of NAc between MDD and normal group using the Hodgkin-Huxley model (H-H model) (Li et al., 2022). This provided the theoretical basis for this article. Here, we will still use the H-H model, but with the dynamical receptor binding model for computation, to explore the relations among MDD, dopamine concentrations, and VTA-NAc-mPFC neural circuits. The simulation results replicated some important pathological patterns of MDD at the level of neurons and circuits with low dopamine concentration and gave some explanations for the regulation effects on membrane potentials, ion channels, synaptic activities, and local field potentials. It was the first computational model to illuminate the effect of dopamine concentrations for the NAc-mPFC-VTA circuit between MDD and normal groups, which can be used to quantitatively explain the results of existing physiological experiments, predict the results for unperformed experiments, and screen possible drug targets.

## Materials and Methods

### Network Structure

**FIGURE 1:**
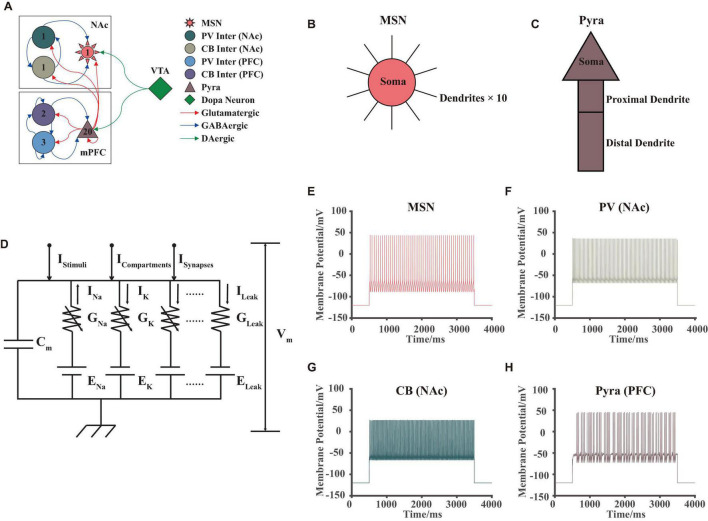
VTA-NAc-mPFC neural network model. **(A)** The structure and synaptic connections of the neural network, different colors, and shapes corresponded to different types of neurons; numbers inside the shapes showed the amounts of the neurons in our model; the red, blue, and green arrows showed the glutamate, GABA and dopamine projections, respectively. **(B,C)** Structures of the MSN in NAc and pyramidal neurons in mPFC in our model, where the 10 dendrites of the MSN were the same. **(D)** The circuit schematic of the H-H model for an individual neuron, whose currents consisted of 4 different types: ionic currents I_*Ions*_, stimuli current I_*Stimuli*_, compartment currents I_*Compartments*_, and synaptic currents I_*Synapses*_. **(E–H)** Simulated membrane potential results of different types of neurons in our model, the stimuli began at 500 ms and ended at 3,500 ms, which successfully replicated the fast-firing pattern of interneurons and burst firing pattern of pyramidal neurons.

In our model, we simulated some brain regions in the reward and emotion neural pathways, which included three brain regions: NAc, mPFC, and VTA (Figure 1A). NAc included D2-type GABAergic MSNs, PV, and CB types of interneurons. mPFC included D1-type pyramidal neurons, and PV and CB types of interneurons. Pyramidal neurons were responsible for releasing excitatory glutamate while interneurons released GABA, both of them were important for the excitatory-inhibitory balance. VTA consisted of dopaminergic neurons, these types of neurons can synthesize dopamine and release it to other brain regions *via* neuronal projection. We referred to some published articles (Durstewitz et al., 2000; Wolf et al., 2005; Russo and Nestler, 2013; Konstantoudaki et al., 2014; Du et al., 2017), and finally determined the structure of this network as shown in Figure 1A (please see Supplementary Materials for details). Note that in our model, the number of neurons showed differently for different brain regions and neuron types, and this setting was aimed to match the published experimental results: for example, the ratio of the number of pyramidal neurons to the interneurons in the mPFC was generally set to 4:1 (Bazhenov et al., 2004).

Each neuron had its own unique physiological structure. For example, MSNs had a complex dendritic spine structure (Wolf et al., 2005; Du et al., 2017; Lindroos et al., 2018), while pyramidal neurons had a basal dendrite—soma—proximal dendrite—distal dendrite structure (Wang et al., 2004). In this article, we simplified the above-mentioned structures for calculation. About the MSNs, we considered that it consists of 1 soma and 10 same dendritic spine structures (Figure 1B). For pyramidal neurons, we established the soma—proximal dendrite—distal dendrite structure without considering its basal structure (Figure 1C).

Additionally, we did not consider the specific membrane potential changes of dopaminergic neurons in the VTA but considered the different dopamine concentration gradients as a direct input to the model. Thus, the model can reveal the effects of the abnormal dopamine concentration more directly on the NAc-mPFC networks in MDD. And it could substantially improve the calculation speed of our model.

### Hodgkin-Huxley Model

We used the H-H model to simulate the membrane potential for an individual neuron (Hodgkin and Huxley, 1952), where different types of neurons in the above brain regions corresponded to different electronic circuit structures. The basic schematic of the H-H model was shown in Figure 1D. Here, we will not perform additional theoretical derivation and interpretation of this model, since this model had been proved many times in the published article (Hodgkin and Huxley, 1952). Note that in our model, the currents consisted of four main components as ion channel currents, compartment currents, synaptic currents, and stimuli currents. Solving this dynamic circuit model by Kirchhoff’s Law numerically, the ion channel currents and membrane potentials at different moments can be obtained. The differential model was given by the following equation:


(1) $$C_{m}\,\frac{d\,V_{m}}{d\,t}+\sum I_{i\,o\,n}+\sum I_{C\,o\,m\,p\,a\,r\,t\,m\,e\,n\,t}+\sum I_{S\,y\,n\,a\,p\,s\,e}=I_{S\,t\,i\,m\,u\,l\,i}$$

### Ion Currents

The calculation methods for ion channel currents included two main categories (Wolf et al., 2005). One was the currents including sodium, potassium, and chloride ions, which can be calculated by the following equation.


(2) $$I_{I\,o\,n}=G_{I\,o\,n}\,m^{x}\,h^{y}\,\left(V_{m}-E_{z}\right)$$

where *G*_*Ion*_ was the maximum conductance of the ion channel and *E*_*z*_ was the reversal potential. *m*, *h* corresponded to the “activating” and “deactivating” gate states of ion channels. After fitting the tail currents of the ion channels with the Boltzmann function, we can obtain some parameters as the time constant, half-activation voltage, and the gradient, which *m*, *h* can be calculated as follows:


(3) $$\frac{d\,m}{d\,t}=\frac{m_{\infty }-m}{\tau_{m}},m_{\infty }\,\left(V_{m}\right)=\frac{1}{1+\exp\left(\frac{V_{m}-V_{1/2}}{k}\right)}$$

Those parameters all came from electrophysiological experiments or published computational models (Destexhe et al., 1998; Durstewitz et al., 2000; Wang et al., 2004; Wolf et al., 2005). In addition, for an ion channel without a deactivation state (e.g., KIR channel in MSN), we can let y = 0 in Equation (2). And for a partially deactivating ion channel (e.g., KAs channel in MSN), we used Equation (4) to calculate it, where the partially deactivating rate *a* ranged from 0∼1.


(4) $$I_{I\,o\,n}=G_{I\,o\,n}\,m^{x}\,\left(a\,h-\left(1-a\right)\right)\,\left(V_{m}-E_{z}\right)$$

For the other types of ion channels, especially the calcium currents, their ionic currents were not only related to the membrane potentials, but also to the concentration and permeability of calcium ions inside and outside the cell membrane. We referred to the published articles and calculated them using the Goldman-Hodgkin-Katz (GHK) Equation as follows (Heinemann et al., 1992).


(5) $$I_{C\,a^{2+}}=P_{C\,a^{2+}}\,z^{2}\,\frac{V_{m}\,F^{2}}{R\,T}\,\frac{{\left[C\,a^{2+}\right]}_{i\,n}-{\left[C\,a^{2+}\right]}_{o\,u\,t}\,\exp\left(-z\,F\,V_{m}/R\,T\right)}{1-\exp\left(-z\,F\,V_{m}/R\,T\right)}$$

And the concentration and permeability of calcium can be calculated by those two equations. Here, *z* = 2, *F* = 96, 489 C/mol, *R* = 8.31 J/Kmol, T=35⁢C°, [*Ca*^2+^]_*in*_ = 0.001 mM, [*Ca*^2+^]_*out*_ = 5 *mM*. $P_{Ca^{2+}}$ can be approximated with Equation (6), where $\bar{P_{C\,a^{2+}}}$ is the maximum permeability and *m*, *n* are different ion channel gates.


(6) $$P_{C\,a^{2+}}=\bar{P_{C\,a^{2+}}}\,m^{x}\,n^{y}$$

The changing calcium parameter [*Ca*^2 +^]_*in*_ can be calculated as the following equation:


(7) $$\frac{d\,{\left[C\,a^{2+}\right]}_{i\,n}}{d\,t}=k\,\frac{-I_{C\,a^{2+}}}{2\,F\,d}-p\,\frac{K_{t}\,{\left[C\,a^{2+}\right]}_{i\,n}}{{\left[C\,a^{2+}\right]}_{i\,n}+K_{d}}+\frac{{\left[C\,a^{2+}\right]}_{i\,n,\inf}-{\left[C\,a^{2+}\right]}_{i\,n}}{\tau_{R}}$$

Where $I_{Ca^{2+}}$ is incoming calcium current, *d* = 0.1 μ*m*, *K*_*t*_ = 10^−4^*m*M ⋅ (*ms*)^−1^, *K*_*d*_ = 10^−4^*m*M, τ_*R*_ = 43 *ms*, *k* = 1000, *p* = 0.02.

In this article, we used 15, 8, 3, and 6 types of ion channels to simulate MSN, pyramidal neurons, PV interneurons, and CB interneurons. The Kinetic for each ion channel was described clearly. For detailed parameter settings please see Supplementary Information.

Particularly, in our model, we only considered voltage-gated ion channels. We did not consider other complex ion channels, such as pressure-sensitive, temperature-sensitive, and pH-sensitive types.

### Compartment Currents

The compartment current was the simulation of the transmission process of neuronal electrical signals over dendrites and axons, which was used to model the passive membrane property, such as the dendritic integration process. We used Rall’s cable model and compartment model to calculate it (Rall, 1962), considering the soma, axons, and dendrites as different compartments. As an example for an individual MSN, whose soma was connected with the complex dendrites, the compartment current of the soma receiving from the dendrite can be calculated by the following equation.


(8) $$I_{C\,o\,m\,p\,a\,r\,t\,m\,e\,n\,t}=\frac{d_{S\,o\,m\,a}\cdot d_{D\,e\,n\,d\,r\,i\,t\,e}^{2}}{r_{L}\cdot l_{S\,o\,m\,a}\cdot \left(d_{S\,o\,m\,a}^{2}\cdot l_{D\,e\,n\,d\,r\,i\,t\,e}+d_{D\,e\,n\,d\,r\,i\,t\,e}^{2}\cdot l_{S\,o\,m\,a}\right)}$$

where *r*_*L*_ was the axial resistance of the membranes, *d*, *l* were the diameter and length of the compartment, respectively. Similarly, other compartment currents can be calculated based on this method. Because only MSN and pyramidal neurons in this article had complex compartment structure (MSN: soma—dendrites, pyramidal neurons: soma—proximal dendrite—distal dendrite), the compartment model was only used for these two neuronal types.

### Synapse Models

In this article, we focused on modeling AMPA, NMDA, and GABA_*a*_ currents. The receptors corresponding to these three synaptic currents all belonged to ligand-gated receptors. We referred to Destexhe’s published work (Destexhe et al., 1998), who successfully modeled and verified these above three currents using non-linear dynamical methods. For AMPA and GABA_*a*_ currents, they can be calculated as follows.


(9) $$I_{A\,M\,P\,A/G\,A\,B\,A_{A}}=G_{A\,M\,P\,A/G\,A\,B\,A_{a}}\cdot r\cdot \left(V_{m}-E_{A\,M\,P\,A/G\,A\,B\,A_{a}}\right)$$

(10) $$\frac{d\,r}{d\,t}=\alpha \,\left[T\right]\,\left(1-r\right)-\beta \,r$$

where [*T*] was the concentration of the transmitter in the synapse, which varied at different moments. It was determined by the potential of the presynaptic membrane and the maximum concentration of the transmitter. We used the following Equation to calculate [*T*].


(11) $$\left[T\right]=\frac{T_{\max}}{1+\exp\left(-\left(V_{p\,r\,e}-V_{p}\right)/K_{p}\right)}$$

For the NMDA current, since it was subjected to the block of the magnesium ions, *B*(*V*_*m*_) was added to the model so as to match this physiological property (Destexhe et al., 1998).


(12) $$I_{N\,M\,D\,A}=G_{N\,M\,D\,A}\cdot r\cdot B\,\left(V_{m}\right)\cdot \left(V_{m}-E_{N\,M\,D\,A}\right)$$

(13) $$B\,\left(V_{m}\right)=\frac{1}{1+\exp\left(-\left(V_{m}+15\right)/16.3\right)}$$

Additionally, because the strength of the synaptic connection between different neurons was random, we gave a matrix of random numbers in the range (0,1) to model it with a uniform distribution method. Thus, for the postsynaptic neuron *a*_*i*_, if its presynaptic neurons were *a*_*j*1_, *a*_*j*2_, *a*_*j*3_,…, then the synaptic current can be calculated as follows.


(14) $$I_{S\,y\,n\,a\,p\,s\,e,a_{i}}=\sum_{k=1,2,3,\ldots }\omega_{i\,j_{k}}\cdot I_{S\,y\,n\,a\,p\,s\,e,j_{k}\to i}$$

### Stimulus Currents

*In vitro*, researchers usually clamped at different particular currents to study changes in the membrane potential (Perkins, 2006). This was different from the case *in vivo* because the stimulus currents of different neurons were significantly random and varied every time in the networks, even the whole brain. Therefore, in our model, the stimulus currents were random and continuously varied. Since a normal distribution may have some outliers which can be much bigger or smaller than the mean value, the stimulus currents in our model were finally considered to be uniformly distributed within an interval, i.e., *I*_*stimuli*_ ∼ *U*[min, max].

### Parameters Set

The parameter settings of the individual neuron models in this article were mainly referred from published computational articles, which were validated by electrophysiological and molecular biological experiments. The model of MSN came from Wolf’s work (Wolf et al., 2005), who built and simulated complex dendritic structures and ion channels. The models of pyramidal neurons and interneurons (including PV and CB types) came from Wang’s work (Wang et al., 2004). The parameters of the dynamical receptor binding model came from Destexhe’s work (Destexhe et al., 1998). After that, we simplified and adjusted some parameters of these above models in order to match the experiments of MDD and reduce the computational time. The detailed model parameter settings were shown in Supplementary Information.

### Dopamine Input

One Durstewitz’s study provided ideas for our model building (Durstewitz et al., 2000), he successfully built a computational model that can describe the characteristics of membrane potentials in PFC under different dopamine concentrations. In his model, he adjusted the parameters of the high-voltage-activated calcium channel, slowly inactivating the potassium channel, and persistent sodium channel of the pyramidal neurons in PFC, and he studied the synchronization mechanism of this biological network.

However, since this work only calculated the effects under 0% (no dopamine input) and 100% (with dopamine input) conditions of dopamine concentration input, it cannot well demonstrate the effects of continuous, randomly varying dopamine concentration gradients on the PFC. In our model, we considered the dopamine concentration can linearly change the parameter values: if the value of the parameter was *a* in the 0% condition and *b* in the 100% condition, then the value in the *k*% condition was set to *a* + (*b*−*a*) ⋅ *k*%.

Therefore, as for our model in this article, we adjusted the values of some parameters to simulate the membrane properties of MDD and normal groups under different dopamine concentrations. As for pyramidal neurons in mPFC, we changed the maximum conductance of calcium channels (Ca and CaN channel) of soma and distal dendrite, maximum conductance of slowly inactivating potassium channel (KS channel), and the activating and deactivating parameters of the persistent sodium channel (NaP channel) of proximal dendrite according to the dopamine concentrations. As for MSN in NAc, we changed the maximum conductance of the slowly A-type potassium channel (KAs channel) and Ca_*v*_1.2 calcium channel (HVA L-type, CaL1.2 channel) of the soma and dendrites according to dopamine levels. The detailed model parameter settings were shown in Supplementary Information.

Note that because we did not consider the synaptic plasticity in our model, we had not considered the direct modulation effects of dopamine concentrations on the synaptic currents. We preferred to study the effect of dopamine on ion channels. In other words, dopamine did not directly alter mEPSC and mIPSC in our model, but indirectly affected EPSC and IPSC through the alterations of membrane potentials.

### Simulation Experiments

To better analyze the results of the model from a statistical point of view, several randomized simulation experiments were conducted. Since the primary objective of this article was to study the dopamine mechanism of the VTA-NAc-mPFC pathway, we calculated the membrane potentials under 4 different random dopamine concentration gradient range levels (Low 0–25%, Medium 25–50%, High 50–75%, and Full 75–100%) to study the relations between lower dopamine concentration and MDD. After that, 5 dopamine concentration levels (MDD 0%, Low 25%, Medium 50%, High 75%, and Normal 100%) were performed to study the abnormal local field potentials in the MDD group. In which, three independent repeated trials were conducted for each dopamine range level. Also, nine independent repeated experiments were conducted, to rigorously demonstrate that the random connection strength matrix and stimulus current were not significant factors, respectively.

The simulations in this article were mainly performed on Windows 10 Enterprise (version: 1511, CPU: i5-6400, RAM: 32GB), MATLAB (R2020a, MathWorks). For each operation of taking random numbers, the repeatability was strictly determined (using the rng function in MATLAB). The numerical calculations of the model were performed using the second- and third-order Runge-Kutta method with a step at 0.02 ms. The computational results of the model were matched with whole-cell membrane clamp experiments, and some of the membrane potential simulations were shown in Figures 1E–H.

### Statistics

The statistics in this article were all means ± SEM unless otherwise noted. To study the differences in means, we used paired or unpaired Student’s *t*-test. To analyze the differences in variance in multiple groups, we used non-repeated or repeated measures one-way ANOVA methods. All of those above results were FDR corrected. In addition, unless otherwise noted, *p* < 0.05. Statistical analyses were performed mainly with MATLAB (R2020a, MathWorks) and Graphpad Prism (8.0.2).

### Spectral Analysis

In this article, the spectral analyses of the membrane potentials and the local field potentials were performed in both frequency and time-frequency. Because in the process of model calculation, we chose a step at 0.02 ms, which was equivalent to sampling the membrane potential at 50 kHz, a very high frequency. Therefore, before performing the spectral analysis, we first resampled the simulated membrane potential at a sampling frequency of 500 Hz (i.e., 2 ms) to obtain the field potential signal data. After that, a fast Fourier transform was performed, and the power spectral densities of the signal from 0 to 250 Hz were obtained for spectral analysis.

We then set the time window to 100 ms and the overlap rate to 50%, using the short-time Fourier transform for time-frequency spectrum analysis. We converted the power spectral densities into the form of dBm/Hz units for statistics, calculated as *P*_*dBm*_ = 10⋅*log*_10_⁡(*P*_*W*_), where *P*_*dBm*_, *P*_*W*_ were the power spectral densities results in dBm/Hz and Watt/Hz units, respectively.

After that, we performed the Student’s *t*-test to get the *p-value* for each frequency point *f*_*i*_(*i* = 1, 2, …, 1024), noted as *p*_*i*_. We specified the significance level α = 0.1. And when *p*_*i*_ < α, we considered the difference to be significant at the frequency *f*_*i*_. In this way, we can clearly quantify the differences in the frequency distribution among different groups by calculating $D\,i\,f\,f\,R\,a\,t\,i\,o=\frac{N_{d\,i\,f\,f}}{N}$, where *N* = 1024 and *N*_*diff*_ was the number of the different frequencies above.

## Results

### Effects of Dopamine on the Membrane Potentials

**FIGURE 2:**
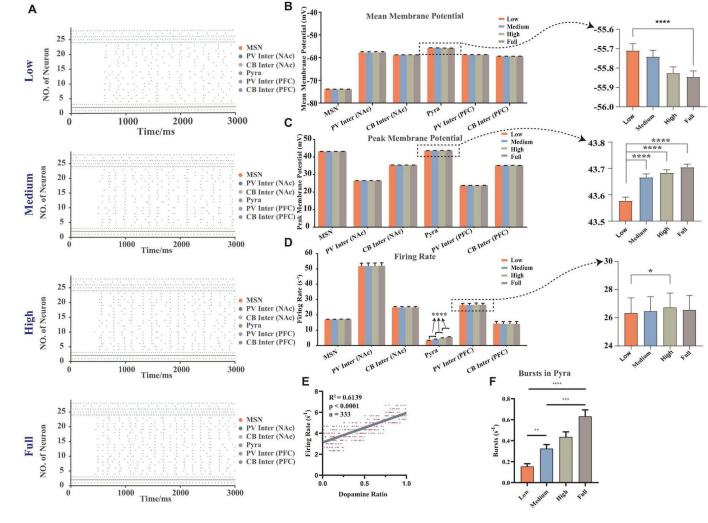
The membrane potentials of different neurons under random dopamine concentration gradients. **(A)** Selected one results of the firing sequence of the action potentials under different dopamine concentrations: low (0–25%), medium (25–50%), high (50–75%), full (75–100%), the strength of connections and stimulus were same (vertical coordinate: neuron number, horizontal coordinate: time, each point showed that the neuron had one action potential at the corresponding moment, different colors were the different types of neurons). **(B)** Statistical results of the mean membrane potentials, which were significantly higher in pyramidal neurons at low concentration than at full concentration, *n* = 180, Brown-Forsythe and Welch one-way ANOVA. **(C)** Statistical results of the mean peak action potentials, when the dopamine concentration increased, the mean peak action potentials in pyramidal neurons significantly increased, *n* = 180, Brown-Forsythe and Welch one-way ANOVA. **(D)** Statistical results of action potential firing rates, when the dopamine concentration increased, the firing rate of pyramidal neurons significantly increased (*n* = 180, Brown-Forsythe and Welch one-way ANOVA), and the firing rate of the high concentration was founded significantly higher than that of low concentration of PV interneuron in mPFC (*n* = 27, repeated measured one-way ANOVA). **(E)** The regression analysis of the firing rates of pyramidal neurons in mPFC with different dopamine concentrations. **(F)** The statistical results of the burst firing of pyramidal neurons, which demonstrated that while the dopamine concentration increased, the frequency of burst firing rose, *n* = 180, Kruskal-Wallis test. All the statistically significant differences were visualized in the graph. *p < 0.05, **p < 0.01, ***p < 0.001, ****p < 0.0001, means ± SEM.

Here we described MDD in terms of dopamine concentrations, with lower dopamine concentrations leading to a greater risk of MDD. The simulated results can accurately replicate the membrane potential results, such as that the firing frequency of pyramidal neurons in mPFC was significantly reduced in MDD mice and decreased in burst firing frequency (Onn et al., 2006), and the membrane potentials of NAc D2-MSNs in MDD mice did not change significantly (Cepeda et al., 2008; Liu et al., 2014; Figure 2). The results revealed that there were some differences in the patterns and characteristics of the membrane potentials of different types of neurons.

We calculated and analyzed the mean membrane potentials (Figure 2B). It was calculated by averaging the membrane potentials over the whole simulation time period. In particular, the reason for analyzing it here was that it was closely related to the local field potential and the subthreshold activity of the individual neuron (Rudolph and Destexhe, 2003), which can demonstrate how the neuron was regulated by synaptic currents in the local microcircuit. The result showed that the dopamine concentrations were significantly and negatively correlated with the mean membrane potentials only of the pyramidal neurons (see the right panel in Figure 2B), and no significant correlations were observed in other types of neurons.

We then analyzed the mean of the peak amplitude of the action potentials (here we named it the mean peak membrane potentials, Figure 2C) and the firing rates (Figure 2D). Results showed that the greatest changes under different dopamine concentration gradient inputs were observed in the pyramidal neurons of mPFC, while those of D2-MSNs in NAc did not differ significantly. It also showed that the mean peak membrane potentials and the firing rates of pyramidal neurons significantly increased while the dopamine concentration rose, which demonstrated that dopamine can excite the pyramidal neurons.

After that, we removed the outlier points of the firing rates of pyramidal neurons (after sorting the firing rates from smallest to largest, only reserving the range of 25–75%), and did a regression analysis with the dopamine ratio (the actual dopamine concentration after adding random variables) as the independent variable and found there was a positive correlation between firing rates and dopamine ratios (Figure 2E). Additionally, we referred to the methods from Hu’s lab to count the burst firing frequency of pyramidal neurons (Cui et al., 2018; Yang et al., 2018). Here, we considered that a burst firing should be counted at the following condition: beginning with a maximal inter-spike interval of 40 ms and ending with a maximal inter-spike interval of 100 ms. Statistical results (Figure 2F) showed that the number of burst firing of pyramidal neurons increased significantly with rising dopamine concentration, which was the same tendency observed in physiological experiments (Onn et al., 2006).

### Ion Currents

We further analyzed the abnormal ion channel current changes. Results showed that the KAs channel of MSNs, KS channel on the proximal dendrite of pyramidal neurons, NaP channel and Ca channel on the soma of pyramidal neurons played an important role in the modulation of this VTA-NAc-mPFC neural network. Compared with the electrophysiological experiments, since our simulation can calculate the currents at every moment, it will better demonstrate the regulation patterns of ion channels under the different dopamine concentration inputs.

**FIGURE 3:**
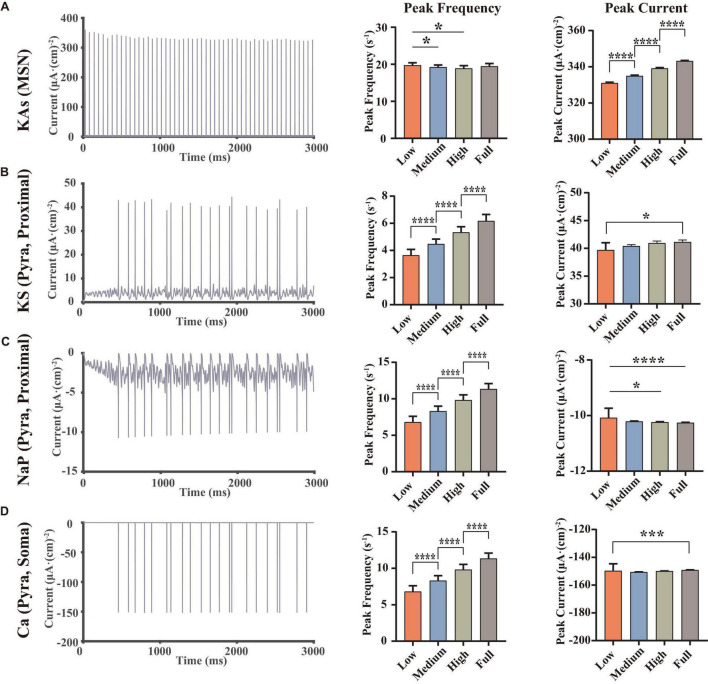
Statistical results of some important ion channel currents, where each row showed a type of the currents [**A**, KAs channel of MSNs, **B**, KS channel on the proximal dendrite of pyramidal neurons. **(C,D)** NaP and Ca channel on the soma of pyramidal neurons, at full dopamine concentration], left, examples for the currents of the corresponding ion channels, middle, statistical results of the peak frequencies (C,D converse), right, statistical results of the peak currents, using repeated measured one-way ANOVA (for normal distribution) or Friedman test (non-normal distributions) methods, *n* = 9. All the statistically significant differences were visualized in the graph. *p < 0.05, ***p < 0.001, ****p < 0.0001, means ± SEM.

From the perspective of the ion channel kinetics, the ionic currents changed mainly in two characteristics: the frequency of the ion currents occurring at the peak (similar to the frequency of the action potentials, here we named it the “peak frequency”) and the amplitude of the peaks (here we named it as “peak current”). Indeed, the peak frequency was determined by the action potential frequency, where each action potential occurring must be accompanied by a peak of ion current; the amplitude of the whole-cell ion current was determined by the conductance and the activation state of the channels, which were related to the microstructure of the ion channels. We did statistical analysis on the peak frequencies and peak currents of the above-mentioned ion channels and analyzed the differences using the repeated measures ANOVA (Figure 3).

The results demonstrated that dopamine significantly increased the amplitude of the peaks of MSN KAs (Figure 3A), while the effect on the peak frequency was not significant, suggesting that dopamine increased the KAs current by affecting the structural characteristics of the KAs channel. In the view of action potential generation mechanisms, the reason for this phenomenon in the MSN KAs channel can be the faster repolarization process. However, because dopamine did not significantly change the mean amplitude of the peak of the MSN’s action potential (Figure 2C), it revealed that the modulation of the KAs channel was not a decisive factor in the MSN membrane potential.

As for pyramidal neurons, we found some significant changes in peak frequency (Figures 3B–D), as well as the increased peak currents of KS (3b) and NaP (3c) channels in Proximal. Since NaP channels were associated with the depolarization state of action potentials and KS channels were associated with the repolarization states, it can be concluded that dopamine can increase the action potential frequency and peaks in pyramidal neurons by modulating those ion channels (Figure 2C). Those simulations replicated the same results reported in some physiological experiments (Yang and Seamans, 1996; Yang et al., 1996), suggesting that NaP and KS channels were the potential target channels that can regulate the membrane potentials of pyramidal neurons efficiently.

In addition, the trend of Ca currents (Figure 3D) in the pyramidal neuron soma was irregular. Since Ca channels were closely related to synaptic activity (Mintz et al., 1995), this suggested that synaptic currents had an important role in regulating action potentials in pyramidal neurons and even local field potentials.

### Synaptic Currents

While activities of ion channels mainly affected neuronal action potential patterns, synaptic transmission activity of neurotransmitters played a more important role to maintain the stability of neural networks, especially the excitation-inhibition balance. Interestingly, Figure 2B showed that the dopamine concentrations were significantly and negatively correlated with the mean membrane potentials of the pyramidal neurons. Also, it was shown in Figure 2D that the firing rates of fast-firing PV interneurons in mPFC were significantly different between low and high dopamine concentrations, yet not in other groups. That evidence suggested that dopamine may change the synaptic currents and the firing patterns of the networks *via* regulating the excitation-inhibition balance.

**FIGURE 4:**
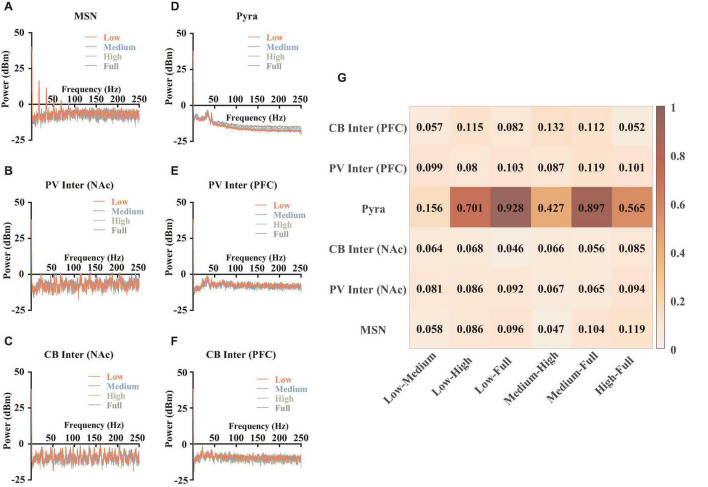
Spectral analysis and statistical results. **(A–F)** Spectral results for different types of neurons, which were averaged over 9 independent replicated experiments. **(G)** The quantifications of the differences. The calculation method was described in section “Materials and Methods” (spectral analysis).

We first performed spectral analysis on different types of neurons, in order to analyze the effects of synaptic currents under the modulation of abnormal dopamine levels in MDD. We resampled the simulated membrane potentials and calculated them separately according to the methods written in section “Materials and Methods” (Spectral analysis), and the results after averaging were shown in Figures 4A–F. It was found that PFC pyramidal neurons showed more significant differences above 50 Hz (Figure 4D), while the power spectral densities of these signals in this range showed a correlation with dopamine concentrations. For other types of neurons, although the time-frequency spectrum differed at different dopamine concentrations, there were no significant regular patterns, implying that the variability of the frequency distribution of those individual neurons was not significant. We quantified the frequency distribution variability (Figure 4G) and found that the pyramidal neuron differences were highly significant.

**FIGURE 5:**
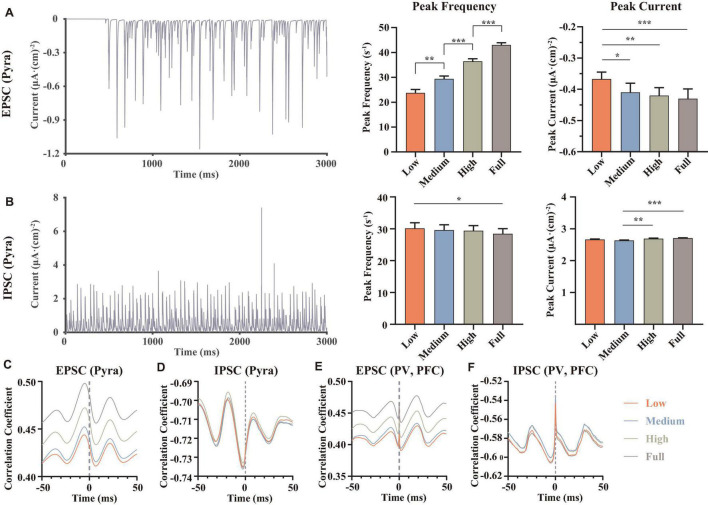
Statistical and correlation results of synaptic currents. **(A,B)** Examples for an EPSC of proximal dendrite and an IPSC of soma both in pyramidal neurons at low dopamine concentration, then we counted the peak frequencies and the mean amplitude of the peaks (named peak current), *n* = 9. **(C–F)** The correlations between the membrane potentials and the synaptic currents of different types of neurons, each result was averaged over 9 independent repeated trials, repeated measured one-way ANOVA (obeying normal distribution) or Friedman test (non-normal distribution), *n* = 9. All the statistically significant differences were visualized in the graph. *p < 0.05, **p < 0.01, ***p < 0.001, means ± SEM.

We further explored the reasons for the differences in the frequencies of pyramidal neurons. We calculated EPSCs (Figure 5A) and IPSCs (Figure 5B) of PFC pyramidal neurons and did statistics. The results revealed that although we set linear dopamine concentration gradients, the significant differences among different dopamine concentrations of EPSCs and IPSCs were not linear. In addition, the peak frequencies and the peak currents of EPSCs in pyramidal neurons both increased significantly, while the peak frequencies of IPSCs tended to decrease. An interesting result was that the peak currents of IPSCs in pyramidal neurons first decreased and then increased (Figure 5B), which was consistent with the trend of Ca current (Figure 3D), suggesting that Ca channels were related to GABA channels. Then, in order to explore the effect of postsynaptic current alteration on postsynaptic membrane potentials, we calculated the correlations between them. The results demonstrated that dopamine can affect and reinforce depolarization of the pyramidal neurons with concentration-dependence (Figure 5C). Also, although there were no significant regular trend in IPSC (5d) results, GABA could still be involved in regulating the stability of the membrane potential under the modulation of excitation-inhibition balance (Seamans and Yang, 2004).

After that, we also calculated the correlations between the membrane potentials and the EPSCs (Figure 5E) or IPSCs (Figure 5F) in PV interneurons of mPFC. The results showed that dopamine also improved the correlations between the EPSCs and the membrane potentials, suggesting that dopamine can affect the whole network to increase the excitations of the glutamate receptors, rather than only regulating some specific glutamate receptors of pyramidal neurons.

### Local Field Potentials

**FIGURE 6:**
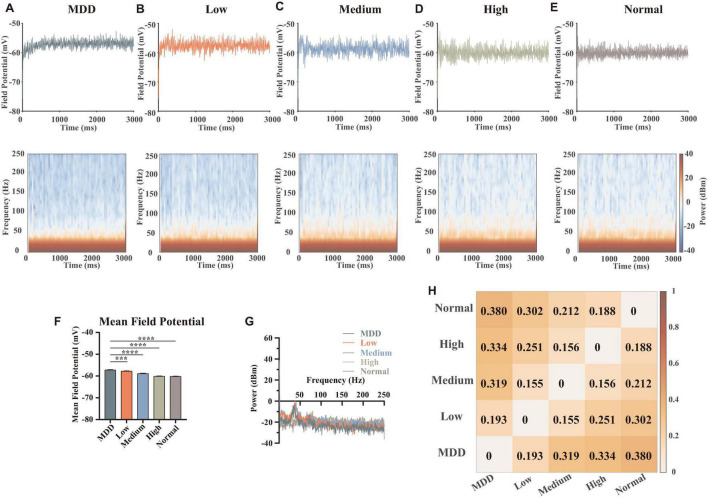
Results and statistics of time-frequency analysis at different dopamine concentrations. **(A–E)** Local field potentials and mean time-frequency results of different dopamine concentrations, MDD (0%), Low (25%), Medium (50%), High (75%), and Normal (100%), with the same connection strengths and stimulus currents (*n* = 6). **(F)** Statistical results of mean local field potential (*n* = 6, Brown-Forsythe and Welch One-Way ANOVA). **(G)** Plots of the mean power spectral densities results of local field potentials for different concentrations of dopamine. **(H)** Heatmap quantification for time-frequency results of different concentrations, calculated as described in section “Materials and Methods” (spectral analysis). All the statistically significant differences were visualized in the graph. ***p < 0.001, ****p < 0.0001 means ± SEM.

In order to explore the effects of the abnormal lower dopamine concentrations input in MDD on the local field potentials of the network, we adjusted the dopamine input in the model. Here, we removed the random components of the dopamine concentration gradients input and added an MDD group. Thus, the dopamine concentration gradients in this part were MDD (0%), low (25%), medium (50%), high (75%), and normal (100%), with a total of 6 replicated trails (the strength of the connections and the stimulus currents were randomized) for each level. We summed and averaged the membrane potentials of the 28 neurons in the network and calculated and plotted the time-frequency heatmaps (Figures 6A–E). Then we counted the mean amplitudes of the peaks in the local field potentials (Figure 6F) and the frequency spectrum (Figure 6G).

We specifically studied the gamma band (30–100 Hz), because the gamma band is associated with the excitation-inhibition balance in the cerebral cortex. In addition, this band is related to the functional properties of PV interneurons and the brain’s ability to integrate information (Bartos et al., 2007; Atallah and Scanziani, 2009; Mann and Mody, 2010). Some evidence suggested that ketamine can modulate the firing patterns of microcircuits in PFC with disinhibition to achieve a rapid antidepressant effect (Seamans, 2008; Miller et al., 2016; Cui et al., 2018; Yang et al., 2018), suggesting an important role of excitatory-inhibitory balance in the regulation of MDD. In this article, due to our simulation results, we found that in the range of 30–50 Hz, higher dopamine concentration had a lower power spectral density level, while in the range of 50–100 Hz (and even to the high frequency 250 Hz), dopamine concentrations were negatively correlated to power spectral density levels. This suggested that dopamine modulated different frequencies of the local field potentials in different ways, which could be determined by different kinds of synaptic currents.

Some published articles demonstrated when external stimuli were input, pyramidal neuron cells were first excited and followed by the release of glutamates, which can excite GABAergic interneurons (especially PV neurons) and generate a gamma oscillation of interneurons to affect pyramidal neurons. Thus, the neural network was synchronized (Bartos et al., 2007; Cardin et al., 2009). Combined with our simulation results, we explained the dopamine mechanism as follows: when dopamine was input because dopamine can promote the release of glutamate of pyramidal neurons (Figure 2D), the concentrations of glutamate projected to PV interneurons of mPFC became higher (Figure 5E), making PV neurons more excitable (Figure 2D). When the dopamine concentration was further increased, PV neurons released large amounts of GABA to inhibit pyramidal neurons, thus ensuring excitation-inhibition balance in the local neural microcircuit (Figures 5D,F). In addition, the quantifications for time-frequency results of different concentrations showed (Figure 6H) that the differences in dopamine concentrations were positively correlated to the differences in its field potential frequencies, indicating that dopamine was an important factor influencing the field potential frequency distributions.

**FIGURE 7:**
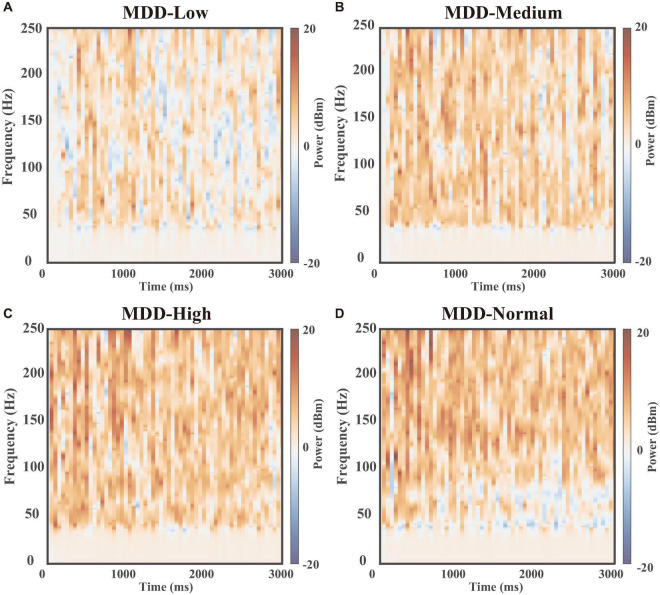
Time-frequency power spectral results. The heat maps of the time-frequency power spectral results of field potentials between the MDD group (dopamine input: 0%) and dopamine groups(dopamine input: **(A)** low 25%, **(B)** medium 50%, **(C)** high 75%, and **(D)** normal 100%).

Subsequently, we displayed the heat maps of the time-frequency power spectral results of field potentials, which can demonstrate the differences between the DA group (Low, Medium, High, and Normal) and no DA group (MDD) at different dopamine gradient levels in the whole neural network (Figure 7), calculated as $\Delta \,P=P_{d\,B\,m,D\,A}-P_{d\,B\,m,C\,o\,n\,t\,r\,o\,l}=10\cdot \log_{10}\left(\frac{P_{W,D\,A}}{P_{W,C\,o\,n\,t\,r\,o\,l}}\right)$. Here, the blue part (−20 to 0 dBm/Hz) showed that the DA group had lower power than the MDD group at the corresponding moments and frequencies, and the red part (0–20 dBm/Hz) showed that the DA group power was higher than that of the MDD group. We found that the high-frequency component of the field potential (100–250 Hz) was significantly enhanced as the DA concentration difference increased, suggesting that DA can improve the firing rate of neurons in the network. Also, we found that Gamma oscillations (30–100 Hz) were stronger in the other groups than in the MDD group in the region of 0–1,000 ms, while the opposite was true in the region of 1,000–3,000 ms (Figure 7D). It demonstrated that high levels of DA can rapidly affect PV interneurons and release GABA to keep the neural network stable, resulting in a synchronized firing rate increase. When the neural network is stable, glutamate will be in charge of maintaining the frequency of action potentials, so the role of GABA decreases, as shown by a gradual decrease in the gamma frequency component over time.

## Discussion

### The Computational Model Can Describe Neuronal Activities at Different Moments

Exploring the dynamically modulated relations of MDD among abnormal membrane potentials, ion channels, and neurotransmitters, especially analyzing these processes quantitatively so as to find the causative factors and therapeutic targets, will be of great significance for early prediction and diagnosis, treatment, and assessment of therapeutic effects. However, even though it was very important for the research of MDD, there were few studies to work on these above-mentioned mechanisms, because the dynamic processes were too complex to be explained by experiments.

The development of computational neuroscience, especially computational psychiatry, has provided the theoretical and mathematical basis for these above problems (Adams et al., 2016; Huys et al., 2016). From the perspective of mathematics, this complex neural system can be considered a dynamic system model: for any given moment, the neural system will have a state which includes biological, physical, and chemical characteristics of membrane potentials, ion channels, receptors, and others (Kringelbach and Deco, 2020). Using the available experimental data on electrophysiology and biochemistry as the inputs and parameters in the models, especially using the data on ion channel kinetics, receptor binding kinetics, and neurotransmitter concentration, we can perform calculations for simulations, predictions, and analysis of the neural system in different conditions.

With these theories, we build a computational model for the NAc-PFC-VTA dopaminergic pathway, based on the results of existing multi-channeled electrophysiological experiments, which can describe the membrane potential changes at different dopamine concentrations. In particular, the abnormal decrease of dopamine concentration is a typical symptom in the brain regions of MDD patients, while our calculated results of low-level dopamine concentration are the same as the electrophysiological results of MDD, such as the significant decrease of PFC pyramidal neuron firing frequency and burst frequency, as well as no significant change of MSN firing frequency (Figures 2B–F). Furthermore, because the parameters in our model came from the experimental data, it demonstrates that our computational model follows biological principles. Therefore, in the model, we use dopamine concentration as a biomarker to distinguish the MDD group from other groups and calculate the membrane potentials under different external stimuli and network connection strengths.

Compared with traditional electrophysiological experiments, computational models have unique advantages. For example, we can calculate the currents of different ion channels and membrane potential at the same moment, which helps us explore the causal relations and the mechanisms of electrophysiological phenomena in terms of the synergistic and antagonistic effects of different ion channels quantitatively. For example, we analyze the synergistic effects of both NaP and KS channels on the proximal dendrites of PFC pyramidal neurons (Figures 3B,C), in order to explain the biophysical mechanism of the significant reductions in firing frequency and a burst frequency of PFC pyramidal neurons in MDD condition.

In addition, model calculations will help us to predict the potential ion channel targets for MDD therapy. For example, we find that the Ca channel of PFC pyramidal neurons is a possible ion channel target, because the peak currents of IPSCs (Figure 5B) in pyramidal neurons first decreased and then increased, which was consistent with the trend of Ca current changes (Figure 3D), while IPSC is reported as a very important and significant role to regulate the excitatory-inhibitory balance in MDD (Seamans and Yang, 2004; Cui et al., 2018). Moreover, although some studies have demonstrated that DA can bind D2 receptors in MSN and thus directly enhance the activity of KAs, our calculations reveal that KAs channels cannot decisively excite the MSN membrane potentials or even the whole neural network, suggesting that KAs is not a key ion channel target for MDD. These calculations cannot only explain some existing physiological experimental results but also suggest some new ideas and provide new perspectives for the mechanism study of MDD.

### Excitation-Inhibition Imbalance in Major Depressive Disorder With Abnormal Membrane Potential and Field Potential Conditions

We perform spectral analysis and time-frequency spectral analysis for neuronal membrane potentials and local field potentials. The calculated results can describe the effect of the abnormal excitation-inhibition balance of MDD in the VTA-Nac-mPFC network. For an individual neuron, EPSC and IPSC together are in charge of keeping the steady membrane potential. We find that dopamine can increase the EPSCs of both excitatory PFC pyramidal neurons and inhibitory PFC PV neurons; and the correlation coefficients between EPSCs and membrane potentials increase rapidly when the dopamine input concentration grows, indicating that EPSCs are important factors in maintaining the excitability of neurons.

Meanwhile, we find that the peak frequencies of IPSC in pyramidal neurons decrease significantly after dopamine administration, indicating that dopamine can decrease IPSCs to increase the excitatory of those neurons; however, the mean amplitude of the peaks in IPSCs increase significantly in pyramidal neurons, indicating that IPSCs under the influence of dopamine can inhibit the membrane potentials. Those two findings are somewhat opposite. Interestingly, we also find there is no obvious pattern in the correlation efficiency between IPSCs and membrane potentials in our calculation results. We haven’t found any published works of literature to explain it. We will design some biological experiments to study this phenomenon.

Local field potentials can describe the excitability and synchronization of the Nac-PFC-VTA neural network. We find that the gamma-band of the field potentials shows significant differences under the influence of different concentrations of dopamine in MDD. Because the gamma oscillations are mainly modulated by the interneurons, it suggests that dopamine can modulate the neural network by affecting GABA and thus the neural network. Furthermore, the computational results of the model suggest that the role of GABA is more important compared to glutamate receptors (AMPA, NMDA) because it can directly modulate the synchronization and stability of the whole neural network. It indicates that GABA receptors can be an important therapeutic target for MDD. This also explains the phenomenon that MDD-like symptoms in mice are attenuated after ketamine inhibits the interneurons through disinhibition effects.

In summary, it is clear that the synergism and antagonism between EPSC and IPSC modulate the excitability and stability of neuronal cells, and their imbalance due to abnormal dopaminergic pathways is one of the important factors that induce MDD. However, we still do not know the exact relations between MDD and excitation-inhibition balance. In other words, which level of excitation-inhibition ratio can be treated for MDD? And what drugs are more effective in regulating the excitation-inhibition ratio? We will gradually explore this in future studies.

### Guide the Designs of Biological Experiments

The calculation results of the model are useful for guiding the design of subsequent MDD experiments. On the one hand, the calculation results can make predictions for the unperformed experiments, explore possible and potential MDD targets, and reduce the experimental cost as much as possible while ensuring the experimental effect. On the other hand, some existing MDD ion channel targets can be quantitatively analyzed. Researchers can select and study the research objects with great influence on membrane potentials or other physiological indicators to increase the success rate of experiments (Adams et al., 2016; Huys et al., 2016).

From the perspective of mechanism explanation, although there are many existing experimental studies on MDD, most of them only consider the influence of a single variable but do not analyze the mechanism of MDD generation from the multivariate and system biology perspective. Whereas using computational models can analyze the synergistic and antagonistic effects of different ion channels and synaptic currents in regulating membrane potential and local field potential, as well as the causal relation of those different changing research objects. In addition, it is useful for researchers to analyze the pathogenesis of MDD from synchronization and stability, which provides a new idea for the subsequent target exploration.

### A New View for Major Depressive Disorder Diagnosis and Drug Treatment

The model we built can provide new assessment tools for MDD diagnosis and drug treatment. For example, the results of field potential time-frequency analysis calculations can describe the frequency distribution at different moments. We can clearly understand at which moments and in which frequency range MDD differs from other groups, which establishes the correlations between field potentials and MDD at the cellular level. Thus, we can assess the severity of the onset of MDD in animals by the model. In addition, compared with large-scale EEG and fMRI experiments, the calculated results of field potential time-frequency analysis accurately demonstrate not only the changes of field potential after drug treatment but also the microscopic changes of ion channels and synaptic currents in the neural circuit after drug administration. We can calculate the time consumption of altering the abnormal field potential time-frequency pattern of MDD to normal in order to find the best doses and drugs. We can also analyze it with other biological experiments to explain the pharmacological mechanism and metabolic kinetic mechanism.

In addition, some published metabolomics results suggest that some metabolites related to energy metabolism are abnormal in the MDD brains, which reveals that MDD symptom is accompanied by abnormal energy consumption in the brain (Abdallah et al., 2014; Yoon et al., 2016; Zuccoli et al., 2017; MacDonald et al., 2019; Li et al., 2022). We have explored the correlation between neural energy and neural activity in a series of past studies (Zhu et al., 2019; Wang et al., 2021; Yuan et al., 2021), which provides a theoretical basis for our exploration of energy income and expenditure in MDD. In the model, we can calculate the energy consumption of electrical activity in the neural network from the membrane potential and ion channels, and synaptic currents at different moments (Wang et al., 2017; Zhu et al., 2018). Since electrical signaling is the core way of information transmission by neurons, the results of electrical activity energy consumption can reveal the ability of neural networks to process biological information under different conditions. It provides a novel perspective to explore the pathogenesis of MDD, and we are in the process of exploring it.

### Limitations of the Current Model

Although we proposed such a valid and novel model, it was built with some deficiencies. Limited by the current experimental techniques and findings (Durstewitz et al., 2000; Cepeda et al., 2008; Russo and Nestler, 2013; Liu et al., 2014), we made some simplifications and assumptions in the model design. For example, according to the results of anatomical and physiological experiments (Russo and Nestler, 2013), there are large cholinergic, calretinin, and somatostatin interneurons in the Nac region in addition to the most important MSNs, PV, and CB interneurons, while the MSNs also has D1- and D2- different phenotypes. However, our Nac model only simulated the most important types (D2-MSNs, PV, and CB interneurons) but understated the other types. The biggest reason for this was that the kinetic characteristics of the other neurons (including ion channels, receptor binding dynamics, etc.) were still unknown and needed to be studied experimentally. Also, biological experiments had not compared the kinetic differences between the same type of interneurons of Nac and mPFC. Therefore, we assumed that they only had synaptic connectivity differences, but no ion channel differences. In addition, we did not consider how presynaptic dopaminergic neurons from the VTA region dynamically modulated dopamine transmitter release through membrane potentials and how they were regulated by feedback from other brain regions. Although we can say that our simplified model could successfully explain the mechanisms of dopamine and MDD from the biophysical view (Durstewitz et al., 2000; Cepeda et al., 2008; Russo and Nestler, 2013), these deficiencies still deserve to be explored further to improve the computational models.

Another thing that should be mentioned here is the selection of the neuron model. At this stage, the modeling of biological neurons diverges into 2 different routes: simplified neuron models and detailed neuron models. The simplified neuron models do not consider the complex structural and dynamical features of neurons but focus on the performance of large-scale neural network clusters in encoding cognitive behaviors, such as the field potential coding theory (Averbeck and Lee, 2004) and the artificial neural network models (Glimcher, 2011; Dabney et al., 2020). In contrast, the detailed neuron models are usually studied for a single neuron, which may contain thousands of dendritic structures (Wolf et al., 2005; Du et al., 2017). Our model lay between these two scales but was closer to the detailed models. On the one hand, we wanted to explore the interrelationships between different ion channels and neurotransmitters, and we therefore needed to preserve the complex dynamical properties. On the other hand, limited by the computational power, we did not model every neuron in every detail, thus we also simplified the neuronal structure. Because of the simplification of the neuronal structures, the firing patterns in this paper could be different from some electrophysiological experimental results *in vitro* and *in vivo* as well as the simulation results of detailed neuron models *in silico*, but this is unavoidable. Nevertheless, we have preserved as many ion channels and structural properties as possible in our models - we would like to simulate this network of 28 coupled neurons with complex ion channels rather than the network with thousands of neurons but of simple neuronal structures, since MDD is correlated with both the network structure and some important ion channels. Our future goal is to expand the number of neurons while bringing the neuronal model as close as possible to the physiological situation, in order to explore the coding patterns of field potentials in MDD.

## Data Availability Statement

The original contributions presented in this study are included in this article/Supplementary Material, further inquiries can be directed to the corresponding authors.

## Author Contributions

YL conceived, designed, performed the experiments, performed network models, conducted the data analyses, and wrote the manuscript. BZ conceived and designed the experiments, revised the manuscript, and helped with the interpretations. XP designed the experiments, helped with the interpretations, and revised the manuscript. YW and XX helped with the interpretations. RW and ZL conceived the experiments, designed the experiments, and revised the manuscript. All authors reviewed and approved the manuscript.

## Conflict of Interest

The authors declare that the research was conducted in the absence of any commercial or financial relationships that could be construed as a potential conflict of interest.

## Publisher’s Note

All claims expressed in this article are solely those of the authors and do not necessarily represent those of their affiliated organizations, or those of the publisher, the editors and the reviewers. Any product that may be evaluated in this article, or claim that may be made by its manufacturer, is not guaranteed or endorsed by the publisher.

## Abbreviations

AMPA: α-amino-3-hydroxy-5-methyl-4-isoxazolepropionic acid
CB: Calbindin interneuron
DA: Dopamine
EEG: Electroencephalogram
fMRI: Functional magnetic resonance imaging
GABA: Gamma-Aminobutyric acid
LFP: Local field potential
MDD: major depressive disorder
(m)EPSC: (miniature) Excitatory postsynaptic current
(m)IPSC: (miniature) Inhibitory postsynaptic current
mPFC: Medial prefrontal cortex
MSN: Medium spiny neuron
NAc: Nucleus accumbens
NMDA: N-Methyl -D-aspartic acid
PV: Parvalbumin interneuron
Pyra: Pyramidal neuron
Som: Somatostatin interneuron
VTA: Ventral tegmental area.
