# Supplementary material for: Dopamine-Mediated Major Depressive Disorder in the Neural Circuit of Ventral Tegmental Area-Nucleus Accumbens-Medial Prefrontal Cortex: From Biological Evidence to Computational Models
Source: Front Cell Neurosci. 2022 Jul 22;16:923039. doi: 10.3389/fncel.2022.923039 (PMC9373714; doi:10.3389/fncel.2022.923039)
Supplement: Supplementary file 1 [file Data_Sheet_1.docx]

**Dopamine-Mediated Major Depressive Disorder in the Neural Circuit of VTA-NAc-mPFC: A Biological Neural Network Model**

Supplementary Materials

Yuanxi Li^1,2^, Bing Zhang^3,4^, Xiaochuan Pan^1^, Yihong Wang^1^, Xuying Xu^1^, Rubin Wang^1,*^, Zhiqiang Liu^3,5*^

**Independent Experiment Settings**

In this paper, two different groups of parameters were set for simulating independent experiments to replicate the results of the biological experiments: Figure 1-5 corresponded to parameter set A, while parameter set B was for Figures 6-7. Parameter sets A and B were identical in all parameters (including ion channel parameters, neuronal structure parameters, and network structure parameters) except for the different settings of stimulus currents input and dopamine concentrations. To ensure the duplication of the experiments, we use random seeds to hold the different random variables (MATLAB). In this way, we minimized the random errors caused by artificial parameter selection, which could bias the experimental results.

**Network Structure**

The neural network model consisted of 28 neurons. Classified by brain regions, it included nucleus accumbens/NAc (consisted of medium spiny neurons/MSN ×1, parvalbumin interneuron/PV ×1, calbindin interneuron/CB×1) and medial prefrontal cortex/mPFC ( pyramidal neuron×20, CB interneuron×2, PV interneuron×3 ). The reason why we set up these neuron numbers are below.

It was confirmed in physiological experiments that 95% of the neurons in NAc are MSNs[[^1^](#_ENREF_1)]. In this region, excitatory projections come from pyramidal neurons in the mPFC, whereas inhibitory projections come from interneurons in the NAc itself. For the mPFC region, it consisted of ~80% pyramidal neurons and ~20% interneurons, where the excitatory projections are from its pyramidal neurons and its interneurons release GABA as inhibitory projections[[^1^](#_ENREF_1)].

However, the NAc region in our model only include 1 MSN, 1 PV interneuron and 1 CB interneuron. This is because if we obey the biological evidence strictly, the MSN would need to occupy 95%, which should be at least 40 neurons in the NAc region including 38 MSNs, 1 PV and 1 CB interneurons (if we consider the ratio of PV to CB is 1:1 and other neuron types don’t exist in our model). However, for our single MSN model, we considered 14 different ion channel types with very complex kinetics activities, which lead to a differential equation with 98 variables when calculating it (To build the MSN’s model, we referred to Wolf’s work[[^2^](#_ENREF_2)], which was a very complex but detailed model). If we were to increase the number of MSNs and consider everything in detail, this would make the calculations so complex and not efficient.

But this does not mean that the setting of the number of neurons in our model is arbitrary. We adjusted the parameters for different synaptic currents in our model to make our model fit biological evidences. In brief, we adjusted the synaptic conductance based on the neuron number ratio. For example, MSN from NAc and pyramidal neurons from mPFC are under the modulation of GABA (here is GABAA). Since there are only 2 interneuron (1 PV and 1 CB) releasing GABA to MSN in NAC, and 5 interneuron(3 PV and 2 CB) releasing GABA to pyramidal neuron in mPFC, we consider adjusting the GABA conductance so that the total GABA conductance is approximately equal (for MSN - 0.3, for pyramidal neurons - 0.1). For the estimation of the initial conductance, we referred to some other computational and experimental articles. According to both, we adjust all the parameters to best values for simulation based on available conductance data and appropriate biological assumption and finally got all the results.

About the neuron type ratio in mPFC, we set up the ratio as 4:1 (pyramidal-interneuron), and finally it has 25 neurons (20 pyramidal neurons, 3 PV interneurons and 2 CB interneurons). Here, we cannot determine the exact ratio of the number of PFC and NAc neurons, which is also difficult to estimate experimentally, so we can only give some assumptions (25 in mPFC vs 40 in NAc). The reason we did not simplify the number of neurons in mPFC is because the computation of these neurons is more simple than that of MSN, thus we don’t want to lose some information. In fact, although we simplify the model, we want to reserve more information and keep the key information in it. Thus, for our model, because our key information is the ion channels, synaptic currents and the different neuron types, we made some assumptions not only about the type and ratios of neuron numbers, but also simplifying the model of MSNs (though each MSN has a different morphology, it would be impossible to be simulated individually for different morphology if a large-scale simulation is performed, thus we considered MSNs has the same dendritic spiny structure).

Based on the published paper[[^1^](#_ENREF_1)], we built the connection strength matrix to describe the level of synaptic connectivity between different neurons. We considered that if there was no synaptic connection between two neurons, then its strength was 0. For all other pairs of neurons with synaptic connections, they obeyed a uniform distribution of [0,1] ($U[0,1]$). Especially, the diagonal elements of this matrix is set to 0 (we didn’t consider self-connection). For the parameter sets A and B, we performed three trials and chose the same random seeds (from 1 to 3). According to the above principles, despite its less-than-perfect setup (e.g., missing dendritic spine structures), we finally built our 28-neuron model, which kept as many key features of the original neural network (connections, neuron proportions) and can be calculated easily.

**Stimulus Currents**

We simulated the effect of randomly stimulus currents on the network. For the same type of neurons, their stimulus current intensities were the same, while the only difference came from the random error. Since the normal distribution can cause some outlier points and we are not sure if the model was strongly robust, this may have a large impact on the model results. Therefore, we still used a uniform distribution model for quantifying the stimulus currents. We referred to the published paper[[^3-5^](#_ENREF_3)] for the electrophysiological experiments and tried different sizes of random current patterns to finally determine the relevant parameters. In both parameter sets mentioned above, the stimulation current patterns were random currents. One trial was conducted for parameter set A (random seed: 26) and two were for parameter set B (random seed: 26~27), respectively. The detailed parameters were as follows:

$$I_{NAc,MSN,soma}\sim U[2,4]$$

$$I_{NAc,MSN,dendrites}\sim U[2,4]$$

$$I_{NAc,PV}\sim U[0.5,1.5]$$

$$I_{NAc,CB}\sim U[0.1,0.5]$$

$$I_{mPFC,pyramidal,soma}\sim U[1,2]$$

$$I_{mPFC,pyramidal,proximal}\sim U[1,2]$$

$$I_{mPFC,pyramidal,distal}\sim U[1,2]$$

$$I_{mPFC,PV}\sim U[0.5,1.5]$$

$$I_{mPFC,CB}\sim U[0.1,0.5]$$

**Dopamine Concentrations**

We improved Durstewitz’s method and work[[^4^](#_ENREF_4)]. We adjusted the dopamine concentrations to simulate the abnormal firing patterns in VTA-NAc-mPFC network of depressed patients.

The dopamine concentration ratios for parameter set A was a randomized value including 3 trials (random seed: 51-53), which were set up as follows.

$$Low\sim U[0,0.25]$$

$$Medium\sim U[0.25,0.5]$$

$$High\sim U[0.5,0.75]$$

$$Full\sim U[0.75,1]$$

In parameter set B, the dopamine concentrations were constant.

$$MDD=0$$

$$Low=0.25$$

$$Medium=0.5$$

$$High=0.75$$

$$Normal=1$$

**Neuron Models**

*Medium Spiny Neuron*

The model was from Wolf's paper[[^2^](#_ENREF_2)]. The Medium spiny neuron consisted of a soma and 10 dendrites. Each dendrites were with identical ion channel and morphological properties. The soma included NaF, NaP, KAf, KAs, KIR, KRP, Leak, BKKCa, SKKCa, CaL1.2, CaL1.3, CaN, CaQ, CaR, CaT ion channels (See Wolf’s paper for detailed explanation of those ion channels). Dendrites had all channels in the soma except KRP channel, but some of those parameters were changed to match the biological characteristics. The dopamine input increased the maximum conductance ($g_{\max}$) of KAs (both soma and dendrites) and the maximum permeability ($\overline{P_{Ca^{2+}}}$) of CaL1.2. The parameter settings are described in Wolf's article and were detailed below.

1. For non-calcium ion channels: Ion type, $g_{\max}$, a, $V_{1/2,m}$, $V_{1/2,h}$, $k_{m}$, $k_{h}$, reversal potential. Use Equation 2~4 in the manuscript to calculate those ionic currents.

NaF, soma, 1.5, nan, -23.9, -62.9, -11.8, 10.7, 50 (nan – not exist)

NaF, dendrites, 0.0195, nan, -23.9, -62.9, -11.8, 10.7, 50

NaP, soma, 4e-5, nan, -52.6, -48.8, -4.6, 10, 50

NaP, dendrites, 1.38e-7, nan, -52.6, -48.8, -4.6, 10, 50

KAf, soma, 0.225, nan, -10, -75.6, -17.7, 10, -90

KAf, dendrites, 0.021, nan, -10, -75.6, -17.7, 10, -90

KAs, soma, (0.0104+0.0104/2*Dopamine_Ratio), 0.996, -27, -33.5, -16, 21.5, -90

KAs, dendrite, (9.51e-4+9.51e-4/2*Dopamine_Ratio), 0.996, -27, -33.5, -16, 21.5, -90)

KIR, both soma and dendrites, 1.4e-4, nan, -82, nan, 13, nan, -90

KRP, soma, 0.001, 0.7, -13.5, -54.7, -11.8, 18.6, -90

Leak, both soma and dendrites, 11.5e-6, nan, nan, nan, nan, nan, -70

BKKCa, both soma and dendrites, 0.001, nan, nan, nan, nan, nan, -90

SKKCa, both soma and dendrites, 0.145, nan, nan, nan, nan, nan, -90

2. For calcium channels: Ion type, $\overline{P_{Ca^{2+}}}$, a, $V_{1/2,m}$, $V_{1/2,h}$, $k_{m}$, $k_{h}$, reversal potential. Use Equation 5~7 in the manuscript to calculate those ionic currents.

CaL1.2, both soma and dendrites, (6.7e-6-6.7e-6*0.2*Dopamine_Ratio), 0.17, -8.9, -13.4, -6.7, 11.9)

CaL1.3, both soma and dendrites, 4.25e-7, nan, -33, -13.4, -6.7, 11.9

CaN, both soma and dendrites, 1.0e-5, 0.21, -8.7, -74.8, -7.4, 6.5

CaQ, both soma and dendrites, 6.0e-6, nan, -9.0, nan, -6.6, nan

CaR, both soma and dendrites, 2.6e-5, nan, -10.3, -33.3, -6.6, 17

CaT, both soma and dendrites, 4e-7, nan, -51.73, -80, -6.53, 6.7

The time constants for each ion channel (tau in equation 3) and the detailed computational methods can be found in Wolf’s paper.

Additionally, the initial membrane potential of MSN soma and dendrites were set to -90 mV. The length and diameter of soma were both 16 ($10^{-6} meter$), while the capacitance was 1 $\mu F\cdot(cm)^{-2}$. The length and diameter of the dendrites were 20 and 2.25, and the capacitance was the same with that of soma. The axial resistance is 100 Ω/cm. According to the Equation 8 in the manuscript, $I_{Compartment}$ can be calculated.

*Pyramidal Neurons*

The model was from Wang's paper[[^6^](#_ENREF_6)]. Pyramidal neurons included a soma, a proximal dendrite and a distal dendrite. Soma had the classic potassium ($K^{+}$) and sodium channel ($Na^{+}$), high-threshold calcium ($Ca^{2+}$) and slow calcium-dependent cationic currents($CaN$) and leak current($Cl^{-}$). Proximal dendrite included a persistent sodium current ($NaP$), a slow inactivating potassium current ($KS$) and leak current. Distal dendrite consisted of a calcium channel ($Ca^{2+}$), a transient A-type potassium current ($A$) and leak current. After dopamine input, some ion channel parameters were changed as following: Ion type, $g_{\max}$, phi, reversal potential.

Ca, soma, (1.5-0.3*Dopamine_Ratio), 1, 120

CaN, soma, (0.025-0.005*Dopamine_Ratio), 1, -20

KS, proximal, (16-8*Dopamine_Ratio), 1, -80

Ca, distal, (0.25-0.05*Dopamine_Ratio), 1, 120

See Wang’s paper for details.

*PV and Cb Interneurons*

The model was from Wang's paper[[^6^](#_ENREF_6)]. The ion channels parameters of PV interneurons (both in NAc and mPFC) were the same. The only difference was the connectivity relations of them in the neural network. The input of dopamine did not change the ion channel properties. The Cb interneurons also followed these rules. For detailed parameters, please refer to Wang’s paper.

**Synaptic Currents**

The calculation for the synaptic currents came from Destexhe’s work[[^7^](#_ENREF_7)]. There were three types of synaptic currents in the manuscript: AMPA, NMDA and GABA_a_, which all belonged to voltage-gated receptors synapses. Use Equation 9~14 in the manuscript to calculate the currents. The detailed parameters were as following.

Synapse type, presynapse, postsynapse, $g_{\max}$, alpha, beta, reversal potential

AMPA, PFC Pyra, PFC PV, 0.003125, 1, 0.2, 0

NMDA, PFC Pyra, PFC PV, 0.003125, 0.072, 0.0067, 0

GABAa, PFC Interneuron(both PV and Cb), PFC PV, 0.075, 5, 0.18, -80

AMPA, PFC Pyra, PFC CB, 0.0003125, 1, 0.2, 0

NMDA, PFC Pyra, PFC CB, 0.0003125, 0.072, 0.0067, 0

GABAa, PFC Interneuron(both PV and Cb), PFC CB,0.075,5,0.18,-80

AMPA, PFC Pyra, PFC Pyra, 0.013, 1, 0.2, 0

NMDA, PFC Pyra, PFC Pyra, 0.013, 0.072, 0.0067, 0

GABAa, PFC Interneuron(both PV and Cb), PFC Pyra,0.1,5,0.18,-80

AMPA, PFC Pyra, MSN, 0.02, 1, 0.2, 0

NMDA, PFC Pyra, MSN, 0.02, 0.072, 0.0067, 0

GABAa, NAc Inter, MSN, 0.3, 5, 0.18, -80

AMPA, PFC Pyra, NAc PV, 0.003125, 1, 0.2, 0

NMDA, PFCPyra, NAcPV, 0.003125, 0.072, 0.0067, 0

GABAa, NAcInter, NAcPV, 0.3, 5, 0.18, -80

AMPA, PFC Pyra, NAc CB, 0.0003125, 1, 0.2, 0

NMDA, PFC Pyra, NAc CB, 0.0003125, 0.072, 0.0067, 0

GABAa, NAc Interneuron(both PV and Cb), NAc CB, 0.03, 5, 0.18, -80

**References**

[1] Russo SJ, Nestler EJ. The brain reward circuitry in mood disorders[J]. Nature Reviews Neuroscience, 2013,14(9): 609-625.

[2] Wolf JA, Moyer JT, Lazarewicz MT, et al. NMDA/AMPA ratio impacts state transitions and entrainment to oscillations in a computational model of the nucleus accumbens medium spiny projection neuron[J]. Journal of Neuroscience, 2005,25(40): 9080-9095.

[3] Du K, Wu Y-W, Lindroos R, et al. Cell-type–specific inhibition of the dendritic plateau potential in striatal spiny projection neurons[J]. Proceedings of the national academy of sciences, 2017,114(36): E7612-E7621.

[4] Durstewitz D, Seamans JK, Sejnowski TJ. Dopamine-mediated stabilization of delay-period activity in a network model of prefrontal cortex[J]. Journal of neurophysiology, 2000.

[5] Konstantoudaki X, Papoutsi A, Chalkiadaki K, et al. Modulatory effects of inhibition on persistent activity in a cortical microcircuit model[J]. Frontiers in Neural Circuits, 2014,8: 7.

[6] Wang X-J, Tegnér J, Constantinidis C, et al. Division of labor among distinct subtypes of inhibitory neurons in a cortical microcircuit of working memory[J]. Proceedings of the national academy of sciences, 2004,101(5): 1368-1373.

[7] Destexhe A, Mainen ZF, Sejnowski TJ. Kinetic models of synaptic transmission[J]. Methods in neuronal modeling, 1998,2: 1-25.
